# Impact of Sophrology on cardiopulmonary fitness in teenagers and young adults with a congenital heart disease: The SOPHROCARE study rationale, design and methods^☆^^☆☆^

**DOI:** 10.1016/j.ijcha.2020.100489

**Published:** 2020-03-03

**Authors:** Johan Moreau, Kathleen Lavastre, Huguette Romieu, Françoise Charbonnier, Sophie Guillaumont, Charlene Bredy, Hamouda Abassi, Oscar Werner, Gregoire De La Villeon, Anne Requirand, Annie Auer, Stefan Matecki, Clement Karsenty, Aitor Guitarte, Khaled Hadeed, Yves Dulac, Nathalie Souletie, Philippe Acar, Fanny Bajolle, Damien Bonnet, Laurence Negre-Pages, Thibault Mura, Maria Mounier, Pierre-Emmanuel Seguela, Julie Thomas, Xavier Iriart, Jean-Benoit-Thambo, Pascal Amedro

**Affiliations:** a Paediatric Cardiology and Pulmonology Department, M3C Regional Reference Centre, Montpellier University Hospital, Montpellier, France; b Paediatric and Congenital Cardiology Department, M3C Regional Reference Centre, Toulouse University Hospital, Toulouse, France; c PhyMedExp, CNRS, INSERM, University of Montpellier, Montpellier, France; d Self-perceived Health Assessment Research Unit, EA3279, Public Health Department, Mediterranean Medical School, Marseille, France; e Paediatric Cardiology Department, AP-HP, Necker-Enfants malades, M3C National Reference Centre, Paris Descartes University, Sorbonne Paris Cite, Paris, France; f Epidemiology and Clinical Research Department, Montpellier University Hospital, Montpellier, France; g Paediatric Cardiology and Rehabilitation Centre, Institut-Saint-Pierre, Palavas-Les-Flots, France; h Paediatric and Congenital Cardiology Department, M3C National Reference Centre, Bordeaux University Hospital, Bordeaux, France

**Keywords:** Sophrology, Congenital heart defect, Exercise capacity, Health-related quality of life, VO2_max_, Relaxation

## Abstract

**Background:**

Recent advances in the field of congenital heart disease (CHD) have significantly improved the overall prognosis. Now more attention is being given to health-related quality of life (HRQoL) and promotion of physical activity. Non-invasive relaxation therapy may be effective in cardiac patients concerned with exercise-induced dyspnoea. The SOPHROCARE randomised trial aims to assess the impact of Caycedian Sophrology on cardiopulmonary fitness in adolescents and young adults with CHD.

**Methods:**

The SOPHROCARE trial is a nationwide, multicentre, randomised, controlled study in CHD patients aged from 13 to 25 years old. Patients will be randomised into 2 groups (8 Sophrology group sessions vs. no intervention). The primary outcome is the change in percent predicted maximum oxygen uptake (VO2_max_) between baseline and 12-month follow-up. A total of 94 patients in each group is required to observe a significant increase of 10% in VO2_max_ with a power of 80% and an alpha risk of 5%. The secondary outcomes are: clinical outcomes, cardiopulmonary exercise test parameters (VE/VCO2 slope, ventilatory anaerobic threshold, oxygen pulse, respiratory response to hypercapnia), health-related quality of life score (PedsQL), physical and psychological status.

**Conclusion:**

After focusing on the survival in CHD, current research is opening on secondary prevention and patient-related outcomes. We sought to assess in the SOPHROCARE trial, if a Sophrology program, could improve exercise capacity and quality of life in youth with CHD.

**Trial registration:**

Clinicaltrials.gov (NCT03999320).

## Highlights

• Exercise capacity in patients with CHD is lower than in the general population.
• Non-invasive relaxation therapy may be effective in patients with dyspnoea.
• Evidence based-medicine on relaxation therapy in the CHD population is poor.
• This trial will assess the impact of Sophrology on exercise capacity in CHD patients.

## 1 Introduction

Recent advances in the field of congenital heart disease (CHD) have significantly improved the overall prognosis, and currently most children with a CHD will reach adulthood [1]. As a result, after focusing on the survival of this population, more attention is being given to health-related quality of life (HRQoL) and promotion of physical activity [2], [3]. Indeed, cardiopulmonary fitness, as measured by the maximum oxygen uptake (VO2_max_), correlates with HRQoL of children and adults with CHD [4], [5].

We showed that the exercise capacity in children with CHD was moderately but significantly impaired, with a mean overall VO2_max_ decline of 2% per year [6]. Indeed, from a large cohort of nearly 800 patients, we found that children with CHD were three times more affected by physical deconditioning than healthy children [6]. Moreover, we recently reported the existence of an impaired pulmonary function in CHD children, even in the absence of cardiothoracic surgery, impacting cardiopulmonary fitness and HRQoL [7]. In the adult CHD population, abnormal pulmonary function is an independent predictor of prognosis [8].

Exercise-induced dyspnea in patients with CHD may be related to heart failure [9], impaired pulmonary function [6], [10], or muscular deconditioning. Those mechanisms usually interact together and may be aggravated by behavioural and psychogenic obstacles to physical exercise, as the level of anxiety in children and adolescents with CHD [11], as well as in their parents [12], is classically higher than in the general population. As a result, many teenagers and young adults with CHD suffer from an unpleasant feeling of exercise-induced dyspnoea, and cumulated with social and family barriers to physical activity, often “remain on the side-line” at school or in their social life [2], [13], [14]. The current guidelines have reinforced the promotion of physical activity in this population [3], [15], as CHD patients who have been physically active since childhood are less likely to become sedentary adults [14]. When physical deconditioning is diagnosed and managed at an early stage in chronic diseases, participation in rehabilitation and education interventions stand as a chance of reducing cardiovascular morbidity and mortality [16]. Nevertheless, the motivation of teenagers and young adults with CHD to participate in structured hospital-based prevention programs remains limited, such as in transition or rehabilitation programs [17], [18].

In such patients, Caycedian Sophrology may be of interest, as this non-invasive relaxation therapy may focus on exercise-induced dyspnoea. Indeed, we recently reported from a controlled randomised trial, that children and adolescents with asthma were significantly improved in terms of lung function, after only one session of Sophrology [19]. From ancient Greek “σωζ” (harmony), “φρɳν” (mind), and “λ○γία (study), Sophrology is the “study of the consciousness in harmony”, and focuses on breathing, e.g. the only automatic vital function that can become conscious at any time. In the 1960’s, Professor Alfonso Caycedo, a Colombian neuro-psychiatrist, described this relaxation therapy as a healthcare philosophy, based on the study of human consciousness and the relation between body and mind. Caycedian Sophrology requires a structured method consisting in very practical physical and mental exercises, using techniques such as concentration, deep breathing, relaxation, visualization, and simple movements. Sophrology has been increasingly used in healthcare as an adjuvant therapy to treat pain and/or anxiety in oncology [20], [21], geriatrics [22], obstetrics [23], [24], and dentistry [25].

To our knowledge, Sophrology has not been investigated in the CHD population. From a general perspective, the impact of adjuvant therapies in the CHD population has not been evaluated with a high level of evidence, despite some positive experience reporting the use of clinical hypnosis in children undergoing transesophageal echocardiography [26]. Yet, non-invasive relaxation therapy may be effective on exercise capacity in patients with congestive heart failure [27]. Although acclaimed by the contemporary population, the level of evidence for such adjuvant therapies remains limited. Therefore, the SOPHROCARE randomised controlled trial aims to assess the impact of Caycedian Sophrology on exercise capacity in teenagers and young adults with CHD. We also intend to evaluate, in this population, the impact of Sophrology on patient related outcomes, such as HRQoL and anxiety.

## 2 Methods

### 2.1 Study design

The SOPHROCARE trial is a prospective, multicentre, randomised, controlled, parallel arm study, with a 12-month follow-up. Participants will be randomly allocated in a 1:1 ratio to either intervention or control group arms, with minimization on centre of inclusion and age groups (13–17 years old vs 18–25 years old). Randomisation numbers will be computer generated and assigned in strict sequence, using a secure, web-based randomisation system (CS RANDOM module, Clinsight Software). Randomisation will be managed by the Clinical Research Unit of Montpellier University Hospital, France, independently from the investigators. All screened subjects will be identifiable throughout the study by a unique subject number.

**Fig. 1:**
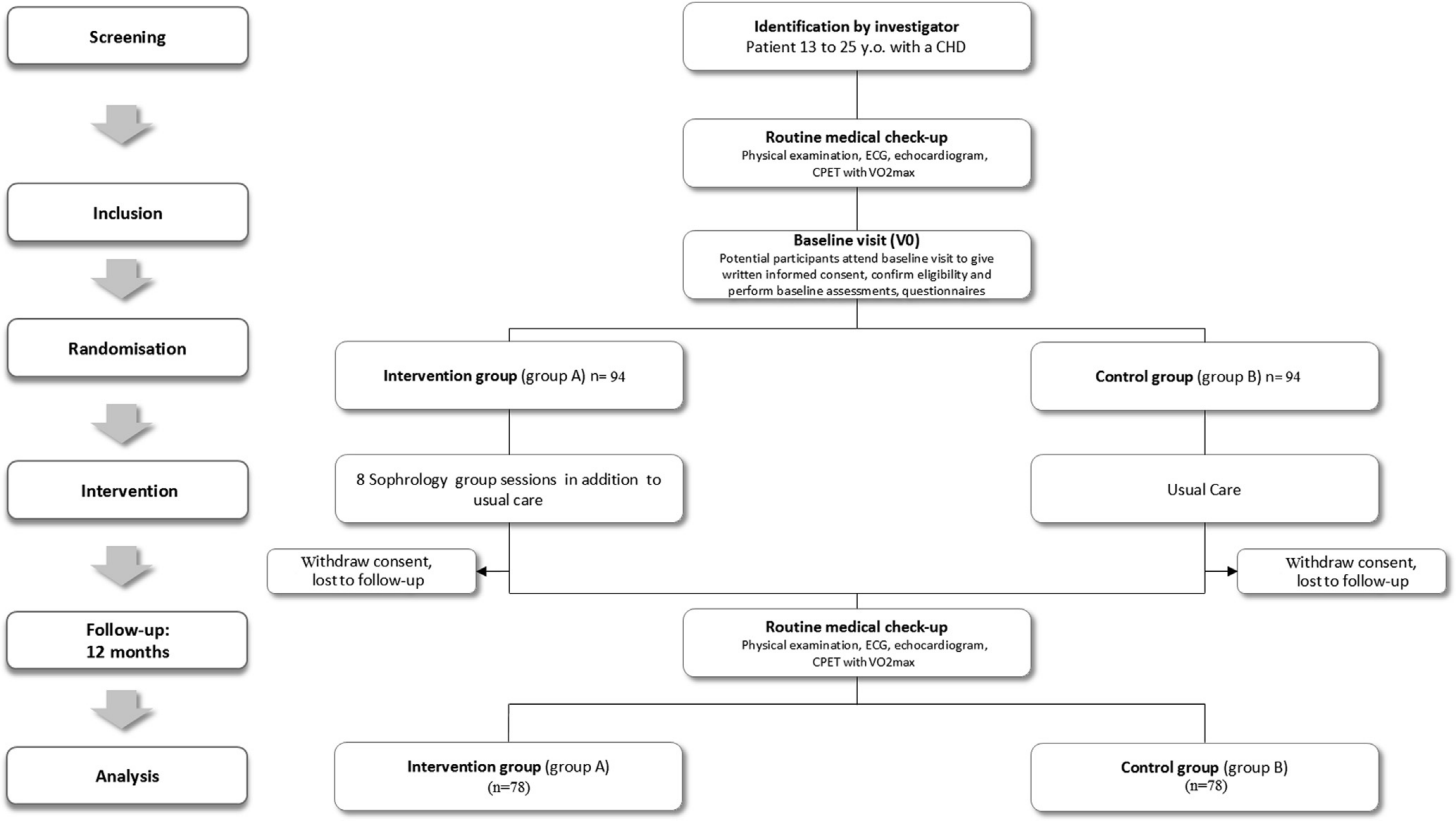

Eligible patients will be randomised into 2 groups (Fig. 1):

• Group 1: intervention group, e.g., patients participating in the Sophrology program in addition to their usual clinical follow-up.
• Group 2: control group, e.g., patients will have a regular non-modified clinical follow-up with no Sophrology intervention during the 12-month study period. However, they will be able to participate in the Sophrology program if they wish, once the 12-month study period is over.

### 2.2 Setting

Overall, 5 CHD centres in France will participate in the study (Montpellier University Hospital, Palavas Saint-Pierre Institute, Toulouse University Hospital, Paris Necker Sick Children University Hospital, Bordeaux University Hospital). Patients will be recruited in tertiary care public institutions, and university centres labelled by health authorities as referral centres for complex congenital heart diseases (M3C national health network). The study will be led by a local principal investigator (supported, when necessary, by a co-investigator), a research nurse or fellow, two sophrologists, and a clinical research assistant, all of whom are trained in Good Clinical Practice and in the requirements of the study protocol. Each site will be responsible for the recruitment and scheduled follow-up visits of participants.

### 2.3 Funding

Montpellier University Hospital is the sponsor of the SOPHROCARE trial. The French National Nursing and Paramedical Clinical Research Program funded this work (PHRIP call for proposal, Ministère des Affaires Sociales et de la Santé).

### 2.4 Study population

Patients with a CHD, as defined by the international ACC-CHD classification [28] and aged from 13 to 25 years old, will be prospectively and consecutively recruited in the participating centres, during an outpatient visit. Patients with a recent (<3 months) medical check-up including a cardiology consultation, an ECG, an echocardiography, and a cardio-pulmonary exercise test (CPET), as detailed in the current European guidelines [29], will be screened (see Table 1).

**Table 1:** Trial entry.

Inclusion criteria
• Male or female aged 13–25 years old
• Patients with a congenital heart disease (CHD), as defined by the international ACC-CHD classification
• Written informed consent for adult patients, or legal guardians for minors

Exclusion criteria
• Absolute contraindication for cardio pulmonary exercise test
• Cardiac surgery or cardiac catheterization planned during the 12-month study period
• Pregnancy in female patients
• Patient undergoing cardiac rehabilitation during the 12-month study period
• Severe intellectual disability with inability to understand the study procedures and/or the quality of life questionnaire
• Expected difficulty to fully participate to the Sophrology program
• Participation in an interventional research during the 12-month study period

### 2.5 Intervention

The SOPHROCARE trial includes a Sophrology program divided into 8 group sessions of 1 h each. All participating centres will use the same program, under the supervision of one of three trained sophrologists (HR, FC, MM), as in our previous similar study [19]. Each group session will be delivered in-person by one of the sophrologists and involve 5 ± 2 patients.

The first part of the session, lasting about 15 min, will start with a discussion (“pre-sophronic dialogue”), in order to create a climate of confidence, including a brief presentation of sophrology. Patients will be asked about their desires, interests and activities. Then the main part of the session, of approximately 30 min, will lead the patient to a level between awakening and sleep (“sophroliminal level”), using a slow and monotone-directed speech. Patients’ state of relaxation should facilitate “letting go,” focusing on body sensations, and improving their well-being (“sophronisation of vital base”). The techniques will be adapted to patients’ age, availability, and energy. The session will end with a final discussion (“post-sophronic dialogue”), putting into words the different sensations felt without any judgment or interpretation (“pheno-description”).

### 2.6 Sample size

A total of 188 patients need to be recruited (94 intervention: 94 control). The primary outcome is the change in percent predicted maximum oxygen uptake (VO2_max_) between baseline (M0) and 12-month follow-up (M12). The mean VO2_max_ from our own cohort of young patients with CHD is 39.4 ± 8.9 ml/kg/min [6]. In the meta-analysis from Gomes-Neto et al., cardiac rehabilitation increases the VO2_max_ by an average of 13% in young CHD patients [30]. In the SOPHROCARE trial, we hypothesized to observe a VO2_max_ increase of 10% in the Sophrology group. Therefore, with an expected difference of 10%, an 80% power, a bilateral alpha risk of 5%, and potentially 20% of loss to follow-up and/or missing data on the primary outcome, we need to include 78 patients in each group. When considering 20% of loss to follow-up and/or missing data on the primary outcome, we need to include 94 patients in the group 1 and 94 patients in the group 2.

### 2.7 Primary outcome

The main outcome is the change in percent predicted VO2_max_ measured during CPET, between baseline (M0) and 12-month follow-up (M12) (see Table 2).

**Table 2:** Outcome measures.

Primary outcome
• Maximum oxygen uptake (VO2_max_)

Secondary outcomes
• Quality of life score: PedsQL self-questionnaire (version 13–18 years for adolescents and version 18–25 years for young adults)
• Proxy version of the PedsQL for parents of adolescents (aged 13–18 years old)
• Level of anxiety (STAI self-questionnaire for young adults and the STAI-Children self-questionnaire for adolescents)
• Level of depression (BDI self-questionnaire for young adults and CDI self-questionnaire for adolescents)
• Other cardiopulmonary exercise tests parameters o Ventilatory anaerobic threshold (VAT) o Ventilatory efficiency (VE/VCO2 slope) o Oxygen uptake efficiency slope (OUES) o Oxygen pulse o Respiratory response to hypercapnia using the rebreathing technique with inhaled CO2 and measuring P0.1 at rest
• Clinical outcomes: NYHA functional class, blood pressure, healthcare usage (primary and secondary care contacts, hospitalisation), and medication
• The socio-economic status of the patient and/or the family (only at baseline)
• Safety outcomes
• Acceptability of the intervention to participants

As in our previous studies, CPET procedures will be harmonised among all participating centres [4], [6], [18]. All centres will use the same CPET cycle ergometer protocol, to obtain a homogeneous incremental overall duration between 8 and 12 min: a 1-minute rest; a 3-minute warm-up (10–20 W) in increments of 10, 15, or 20 W each minute; a pedalling rate of 60–80 rpm; a 3-minute active recovery (20 W); and a 2-minute rest. The CPET will be considered as maximal when 3 out of the 4 following criteria will be reached: respiratory exchange ratio (RER = VCO2/VO2) ≥ 1, maximum heart rate >85% of maximal age-predicted heart rate, limit of the patient's tolerance despite verbal encouragement, inability to provide a minimum pedalling frequency of 60 per minute despite verbal encouragement. VO2_max_ values will be normalized in a percentage of the predicted VO2_max_ using reference values for cycle ergometer test in the general paediatric and adult population [31]. When a plateau of VO2 will not be reached, as commonly observed in children, the peak VO2 (pVO2) will be used [32], [33]. All CPET variables will be centralised and calculated by a single investigator expert in exercise physiology (JM).

### 2.8 Secondary outcomes

The following outcomes will be measured at baseline (M0) and 12-month follow-up (M12) (see Table 2):

• The PedsQL self-reported HRQoL score. Two versions of the PedsQL questionnaire (13–18 and 18–25 years old) will be used for adolescents and young adults, respectively [34].
• The level of anxiety with the self-administered State and Trait Anxiety Inventory (STAI) questionnaire for young adults and the STAI-Children questionnaire for adolescents [35].
• The level of depression with the self-administered Beck Depression Inventory (BDI) questionnaire for young adults and the Child Depression Inventory (CDI) questionnaire for adolescents [36].
• The level of physical activity with the Ricci and Gagnon questionnaire, composed of 8 items (total score of 16 points: no activity; 17–32 points: moderate activity; 33–40 points: intensive activity) [37].
• The clinical outcomes: NYHA functional class, blood pressure, body mass index (BMI), healthcare usage (primary and secondary care contacts, hospitalisation), cardiac events, and medication.
• Other CPET variables: the ventilatory anaerobic threshold (VAT), the ventilatory efficiency (VE/VCO2 slope), the oxygen uptake efficiency slope (OUES), the oxygen pulse (VO2/HR), and the pulse oximetry (SpO2), will be collected, using the same method as in our previous CPET studies [18].
• Respiratory response to hypercapnia using the rebreathing technique with inhaled CO2 and measuring P0.1 at rest [38].

### 2.9 Statistical analysis

A comparative analysis of the baseline characteristics of randomised subjects between the two arms in the study will be performed by giving the frequencies of the different categories for the qualitative variables, and the mean with standard deviation for quantitative variables. Our main analysis will be an intention-to-treat analysis, in which each randomised subject will be analysed in his/her treatment arm. The evolution of the percent predicted VO2_max_ between inclusion (V0) and visit at 12 months (V1) will be compared between the groups using a Student test.

A secondary per-protocol analysis, including all randomised subjects with a valid primary efficacy measurement and with no important protocol deviation (patients who have successfully completed the Sophrology program, with at least 80% of the sessions), will also be carried out to study mechanisms of action. In case of non-comparability of the groups on one of the baseline characteristics, an adjustment will be considered. Analyses will be performed using SAS statistical software (version 9.4; SAS Inc, Cary, North Carolina) using a 5% bilateral alpha risk.

### 2.10 Ethics

The study will be conducted in compliance with the Good Clinical Practices protocol and Declaration of Helsinki principles. It was approved by a drawn National Ethics Committee (North-West I-2018-A00874-51) and registered on Clinicaltrials.gov (NCT03999320). Informed consent will be obtained from all patients and their parents or legal guardians for minors.

## 3 Expected results and perspectives

In the continuity of our research program on exercise capacity in the CHD population [4], [6], [7], [18], [39], the SOPHROCARE trial aims to measure the effect of a non-invasive relaxation therapy, e.g. Caycedian Sophrology, on exercise capacity in teenagers and young adults with CHD. We hypothesized that the efficacy of Sophrology may rely on a global patient management and therefore could improve exercise capacity through different factors.

Firstly, the unpleasant feeling of exercise-induced dyspnoea in patients with CHD has been related to the existence of an impaired pulmonary function with a restrictive pattern in both adults [8] and children [7]. As we recently showed from a randomised controlled trial, Caycedian Sophrology significantly improves pulmonary function in asthma [19]. By analogy, we expect relaxation therapy to improve ventilatory efficiency, classically impaired in the CHD population [6], by focusing on cardiac coherence. To support our physiological hypothesis that Sophrology could improve respiratory drive and therefore ventilatory efficiency during exercise in CHD patients, the SOPHROCARE trial will analyse respiratory response to hypercapnia using the rebreathing technique with inhaled CO2 before and after relaxation sessions.

Secondly, in the SOPHROCARE trial, we purposely focused on a young CHD population, e.g. patients aged from 13 to 25 years old with CHD. This “transition” age group between childhood and adulthood may be at high psychological or behavioural risk [17]. Moreover, youth with CHD are classically overprotected by their parents, stigmatized at school, and often remain on the sideline during sports sessions, despite a normal cardiac condition after surgical repair [2], [13]. Positive mental interactions on physical performance have not been established in this population, who yet may suffer from poor self-image, anxiety, and physical deconditioning [18]. From this perspective, Sophrology may help the patient engage in a process of introspection, improve self-knowledge, provide reassurance, and, as a result, contribute to self-surpassing during physical exercise.

Recent progress in paediatric and congenital cardiology has reduced morbidity and early mortality in the CHD population [1], at the cost of repeated invasive procedures affecting quality of life [2], neurodevelopment [40], and school performance [41]. Therefore, the use of non-invasive mind-body therapies, such as Caycedian Sophrology, is of great interest in young patients with CHD, who may have suffered from anxiety, pain, and psychological stress [42]. From a general perspective, medical progress has been associated with an increasing use of invasive and anxiety-inducing procedures. Moreover, medical sub-specialities mostly focus on a single patient’s organ. In the current era, patients and their families call for more integrated health using comprehensive care [43]. A growing body of evidence supports the effectiveness and safety of mind-body therapies in young patients [19], [44]. Finally, we may expect such non-invasive adjuvant therapies to reinforce the patient-physician bond.

## 4 Conclusion

The multicentre randomised SOPHROCARE trial aims to assess the impact of a relaxation therapy, e.g. Caycedian Sophrology, on cardiopulmonary fitness in teenagers and young adults with a CHD. This study is part of a research program dedicated to improve quality of life and physical capacity of patients with CHD.

## CRediT authorship contribution statement

**Johan Moreau**: Study concept and design, Drafting of the manuscript, Critical revision of the manuscript for important intellectual content. **Kathleen Lavastre**: Administrative, technical, or material support, Critical revision of the manuscript for important intellectual content. **Huguette Romieu**: Study concept and design, Obtained funding, Critical revision of the manuscript for important intellectual content. **Françoise Charbonnier**: Critical revision of the manuscript for important intellectual content. **Sophie Guillaumont**: Critical revision of the manuscript for important intellectual content. **Charlene Bredy**: Critical revision of the manuscript for important intellectual content. **Hamouda Abassi**: Critical revision of the manuscript for important intellectual content. **Oscar Werner**: Critical revision of the manuscript for important intellectual content. **Gregoire De La Villeon**: Critical revision of the manuscript for important intellectual content. **Anne Requirand**: Critical revision of the manuscript for important intellectual content. **Annie Auer**: Critical revision of the manuscript for important intellectual content. **Stefan Matecki**: Critical revision of the manuscript for important intellectual content. **Clement Karsenty**: Critical revision of the manuscript for important intellectual content. **Aitor Guitarte**: Critical revision of the manuscript for important intellectual content. **Khaled Hadeed**: Critical revision of the manuscript for important intellectual content. **Yves Dulac**: Critical revision of the manuscript for important intellectual content. **Nathalie Souletie**: Critical revision of the manuscript for important intellectual content. **Philippe Acar**: Critical revision of the manuscript for important intellectual content. **Fanny Bajolle**: Critical revision of the manuscript for important intellectual content. **Damien Bonnet**: Critical revision of the manuscript for important intellectual content. **Laurence Negre-Pages**: Critical revision of the manuscript for important intellectual content. **Thibault Mura**: Statistical analysis, Critical revision of the manuscript for important intellectual content. **Maria Mounier**: Critical revision of the manuscript for important intellectual content. **Pierre-Emmanuel Seguela**: Critical revision of the manuscript for important intellectual content. **Julie Thomas**: Critical revision of the manuscript for important intellectual content. **Xavier Iriart**: Critical revision of the manuscript for important intellectual content. **Jean-Benoit-Thambo**: Critical revision of the manuscript for important intellectual content. **Pascal Amedro**: Study concept and design, Drafting of the manuscript, Obtained funding, Study supervision, Critical revision of the manuscript for important intellectual content.

## Declaration of Competing Interest

None.
